# Supplementary material for: Evaluation of Nanoparticle-Based Plasma Enrichment on Individuals with Primary and Metastatic Pancreatic Cancer
Source: Cancers (Basel). 2025 Nov 25;17(23):3765. doi: 10.3390/cancers17233765 (PMC12691180; doi:10.3390/cancers17233765)
Supplement: Supplementary file 1 [file cancers-17-03765-s001.zip › cancers-3929631-supplementary.pdf]

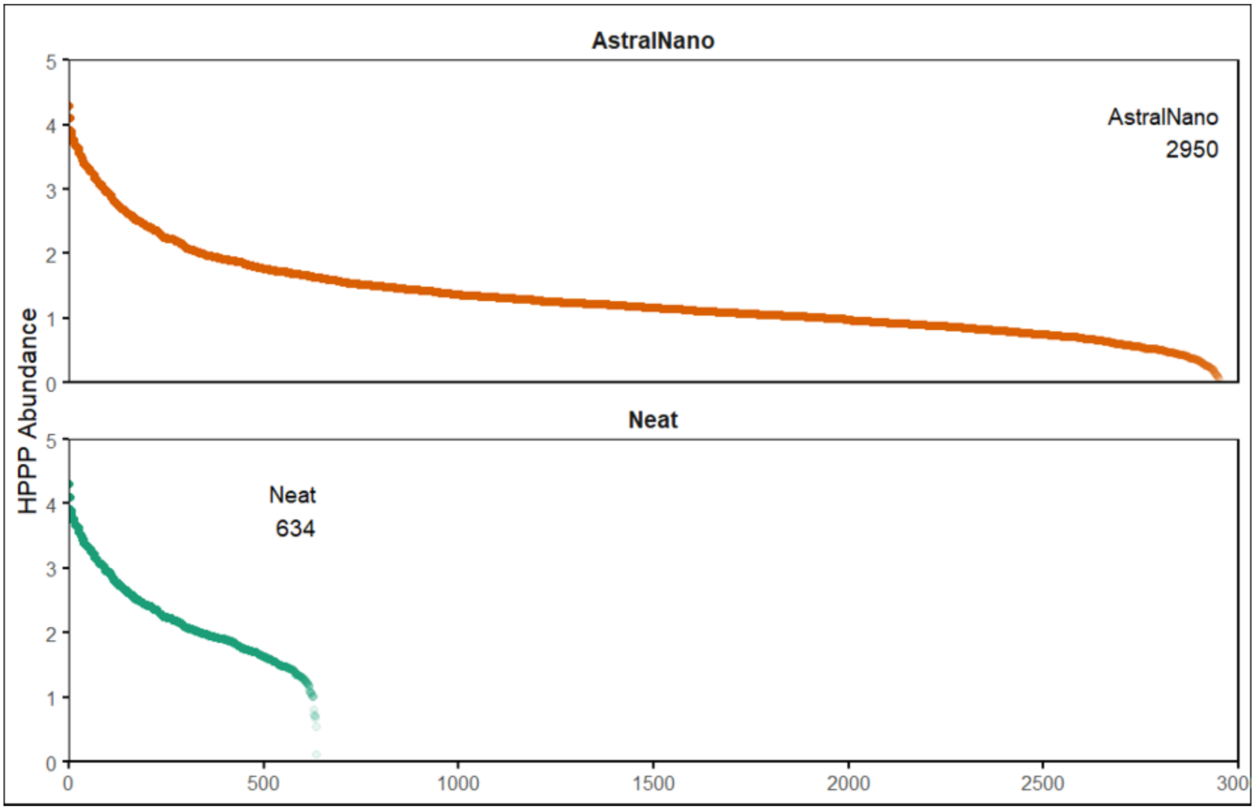

Figure S1. Distribution of proteins identified from nanoparticles enriched (**top**) normal controls and neat digested (**bottom**) plotted onto the HPPP abundance data.

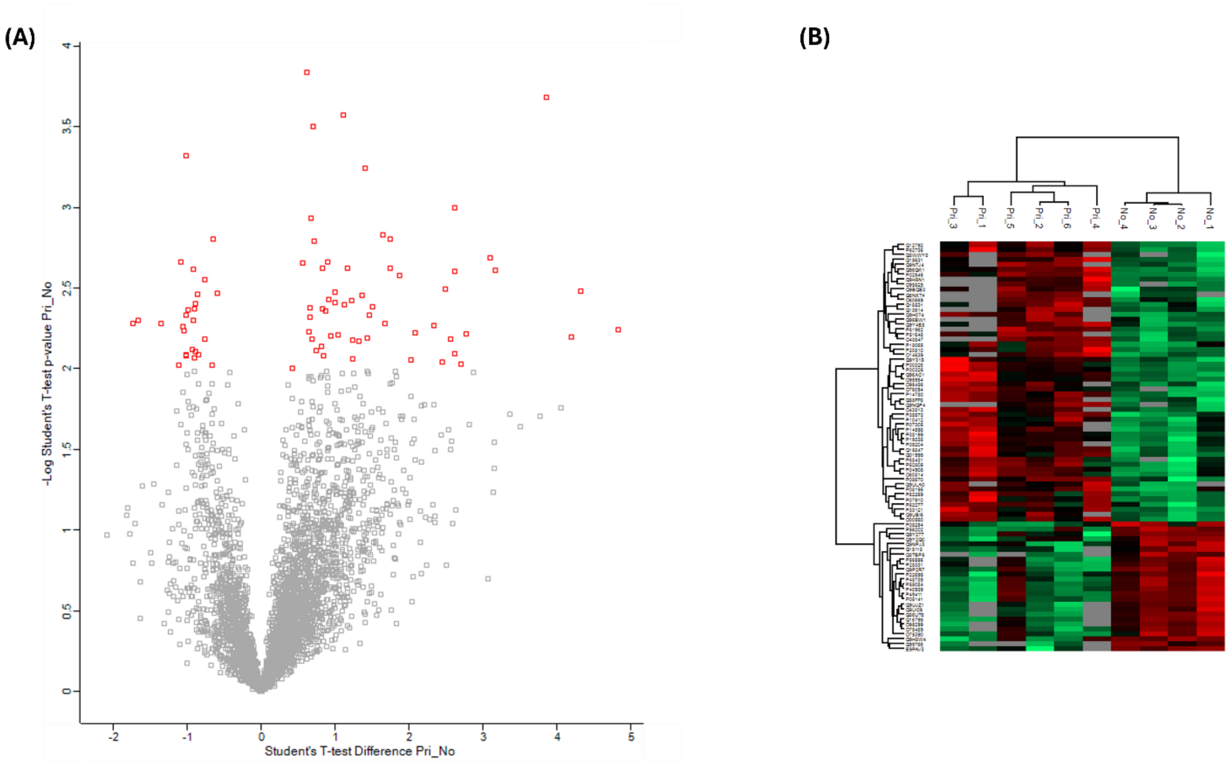

Figure S2. Statistical analysis of individuals with primary PC vs. that of healthy controls. (**A**) Volcano plots were generated based on two-sample Student's *t*-test ($p < 0.01$). Proteins outside the solid curved line are deemed to be statistically significant. Ribosomal proteins (red square) are highlighted. (**B**) A clustering map was generated using Euclidean distance and the complete linkage clustering method with data from all significant proteins (metastasized and healthy control). Each row in the color heatmap indicates a single protein based on the Human UniProt accession number. Normalized protein abundance values (Z-scores) are indicated colorimetrically for each protein as the deviation from the mean by standard deviation units.

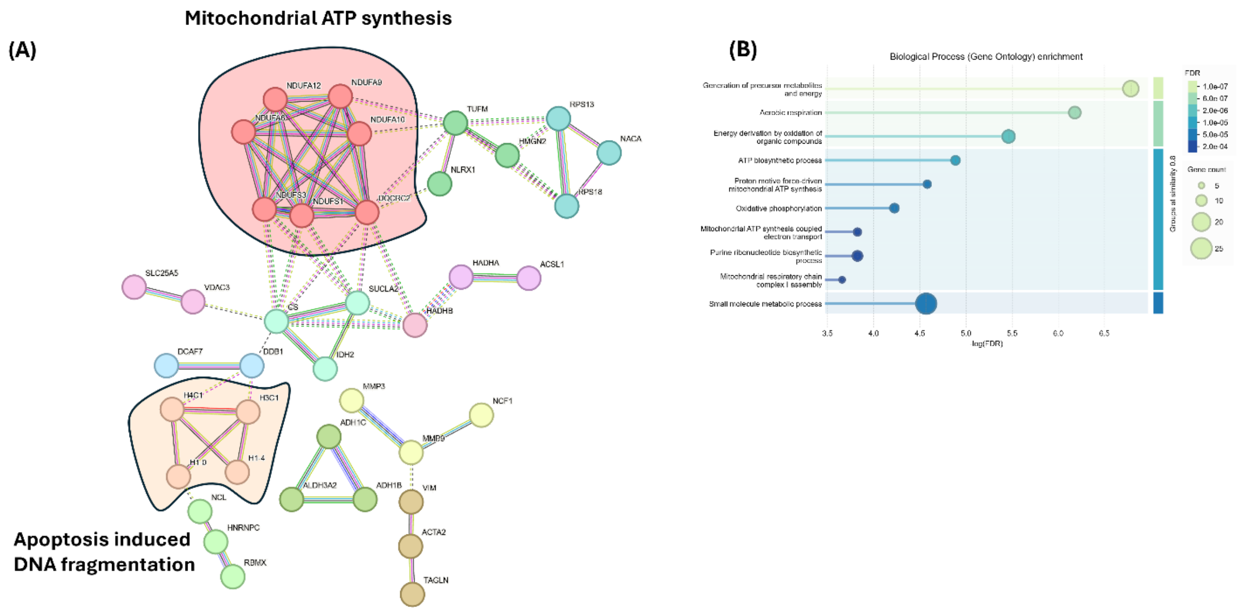

Figure S3. Visualization of known interactions using the STRING database. (**A**) Eighty-two significantly changed proteins of individuals with primary PC vs. that of healthy controls were used in the analysis. (**B**) Four main clusters were revealed from MCL and insert, showing the molecular function (gene ontology) enrichment. PPI enrichment *p*-value = 6.75×10^{-12}.

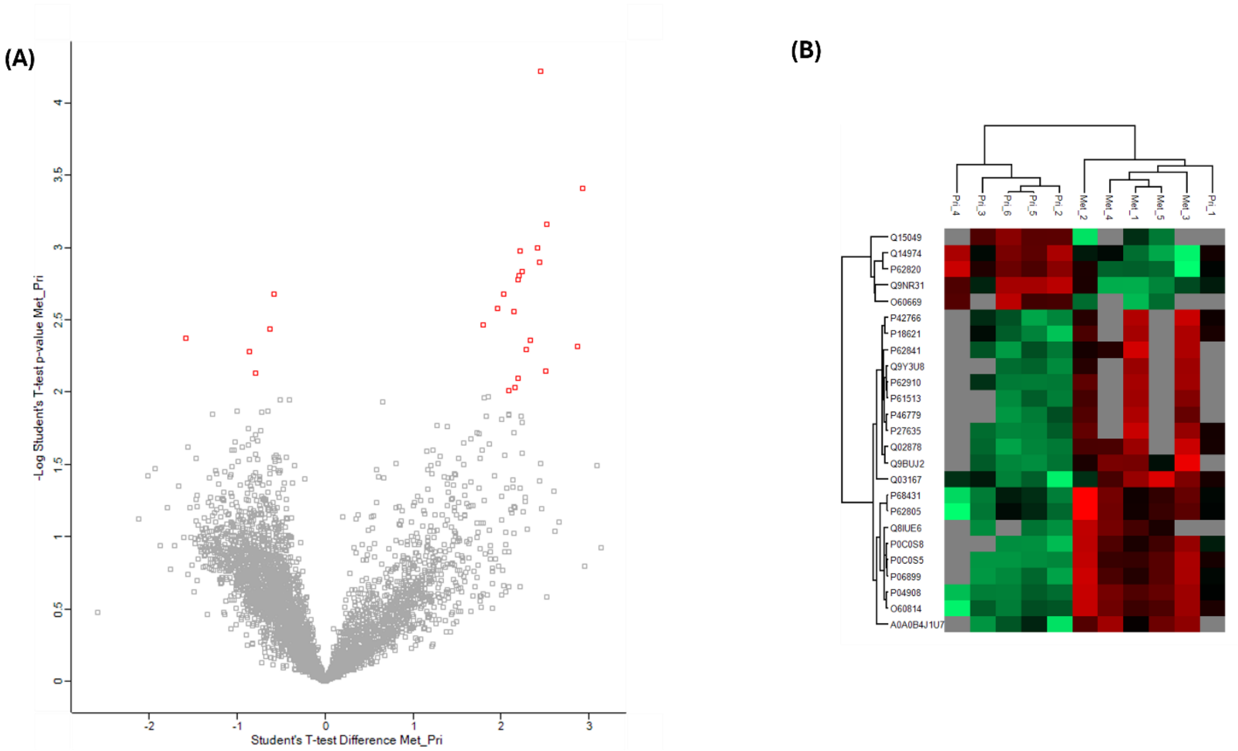

Figure S4. Statistical analysis of individuals with PC that had metastasized vs. primary PC. (**A**) Volcano plots were generated based on two-sample Student's *t*-test ($p < 0.01$). Proteins outside the solid curved line are deemed to be statistically significant. Ribosomal proteins (red square) are highlighted. (**B**) A clustering map was generated using Euclidean distance and complete linkage clustering method with data from all significant proteins (metastasized and health control). Each row in the color heatmap indicates a single protein based on the Human uniport accession number. Normalized protein abundances values (Z-scores) are indicated colorimetrically for each protein as the deviation from the mean by standard deviation units.

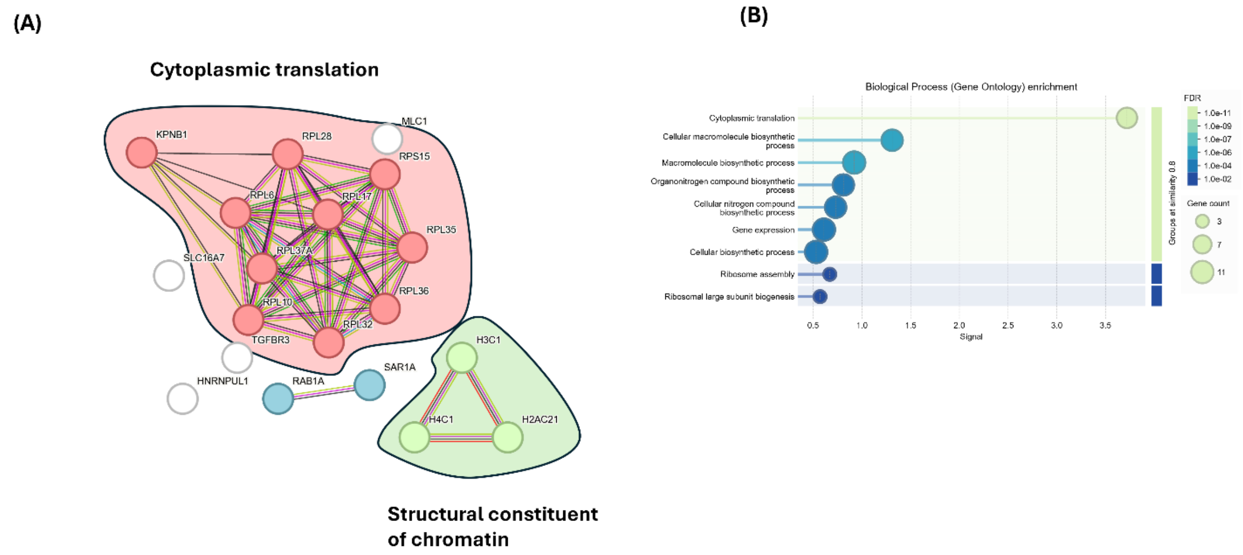

Figure S5. Visualization of known interactions using the STRING database. (**A**) Twenty-five significantly changed proteins of individuals with PC that had metastasized vs. primary PC were used in the analysis. (**B**) Four main clusters were revealed from MCL and insert showing the molecular function (gene ontology) enrichment. PPI enrichment p-value = 3.22 × 10^{-15}.

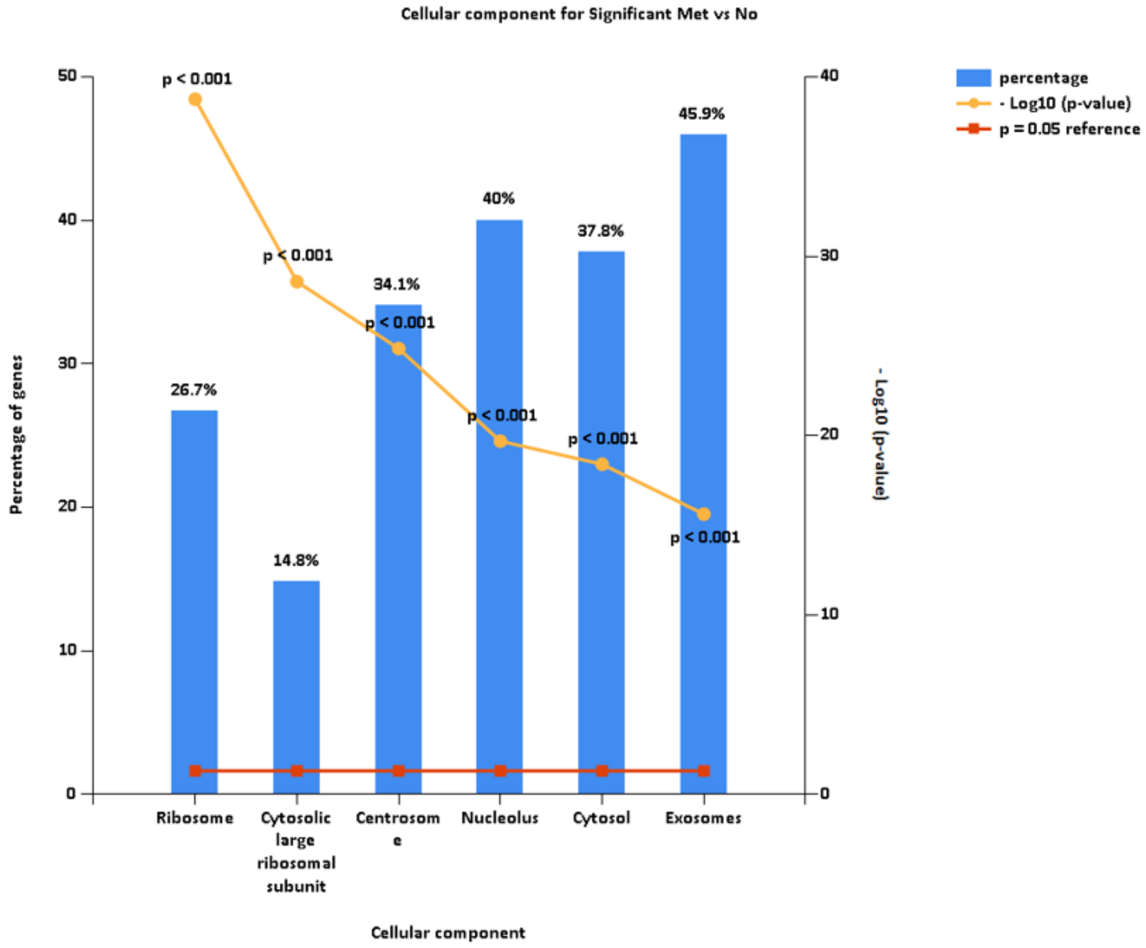

Figure S6. Functional enrichment analysis (cellular components). 140 out of 146 significantly changed proteins in individuals that had metastasized vs that of healthy controls were mapped to the Funrich database [68] and showing exosomes being the largest class of proteins together with the p value from the Hypergeometric test (-Log10 depicted in yellow) and the reference p = 0.05 value (depicted in red).
